# FACILITATORS AND BARRIERS FOR ELDER ABUSE VICTIMS SEEKING HELP: FINDINGS FROM THE NATIONAL ELDER MISTREATMENT STUDY

**DOI:** 10.1093/geroni/igz038.1774

**Published:** 2019-11-08

**Authors:** David Burnes, Ron Acierno, Melba Hernandez-Tejada

**Affiliations:** 1 University of Toronto, Toronto, Ontario, Canada; 2 Medical University of South Carolina, Charleston, South Carolina, United States

## Abstract

Understanding help-seeking among victims of elder abuse is a critical challenge in the field. The vast majority of elder abuse victims remain hidden from formal support/protective response systems, such as adult protective services, legal/justice, law enforcement, or other agencies responsible for addressing this issue in the community. Guided by the Behavioral Model of Health Services Use, this study examined factors that facilitate or impede formal help-seeking among victims of elder emotional, physical and sexual abuse, represented by a call for help in the form of a report to police or other authorities. Data came from a national, population-based elder abuse study in the U.S. with a representative sample (n=304) of victims reporting abuse in the past year. Gold-standard measurement strategies were used to assess each elder abuse subtype. Multivariable logistic regression was conducted to identify help-seeking facilitators/barriers. Help-seeking through reporting to police or other authorities occurred among only 15.4% of elder abuse victims nationwide. Help-seeking was predicted by factors attached to the victim (abuse type, poly-victimization), perpetrator (prior police trouble, social network size), and victim-perpetrator relationship (victim dependence on perpetrator). This study highlights the extremely hidden nature of elder abuse in our society, as well as the need to develop strategies that incorporate victim, perpetrator, and victim-perpetrator relationship factors to promote greater help-seeking among victims.

